# COVID-19–associated delayed-onset MuSK-positive myasthenia gravis presenting solely with respiratory failure: a case report

**DOI:** 10.3389/fimmu.2025.1649930

**Published:** 2025-12-16

**Authors:** Xiao-Guang Cao, Jun-xi Ni, Xiong-feng Zhu, Hua-dong Meng, Chong-jian Huang

**Affiliations:** 1 Department of Emergency Medical Center, The First Affiliated Hospital of University of Science and Technology of China (Anhui Provincial Hospital), Hefei, Anhui, China; 2 Department of Emergency Medicine, The Third People’s Hospital of Hefei, Hefei, Anhui, China; 3 Department of Emergency Intensive Care Unit (EICU),The Third Affiliated Hospital of Anhui Medical University (The First People’s Hospital of Hefei), Hefei, Anhui, China; 4 Suzhou Hospital of Anhui Medical University(Suzhou Municipal Hospital of Anhui Province), Suzhou, Anhui, China

**Keywords:** MuSK myasthenia gravis, COVID-19, respiratory failure, tacrolimus, prognosis

## Abstract

**Background:**

Muscle-specific kinase (MuSK) myasthenia gravis is a rare autoimmune disorder of the neuromuscular junction that predominantly affects the bulbar and respiratory muscles. Although SARS-CoV-2 infection has been implicated as a precipitating factor, post-COVID-19 MuSK-MG remains exceedingly uncommon.

**Case presentation:**

A 29-year-old woman with a six-year history of unexplained exertional dyspnea was hospitalised twice for acute hypercapnic respiratory failure. The first episode, five months earlier, was attributed to severe tricuspid regurgitation; the second occurred eight weeks after mild COVID-19 and was characterized by coma due to hypercapnic respiratory failure without parenchymal lung disease.

**Diagnosis:**

Standard electrophysiology, neostigmine testing and acetylcholine-receptor antibodies (AChR-Ab) were negative. Given persistent ventilator dependence, MuSK antibodies (MuSK-Ab) were measured and found strongly positive.

**Interventions:**

The patient underwent five plasma-exchange sessions, then received oral prednisone (50 mg day), tacrolimus (1.5 mg q12h) and nebulized salbutamol.

**Outcomes:**

She was weaned from non-invasive ventilation by day 23, discharged on day 27 (mMRC grade I) and remained stable on low-dose prednisone/tacrolimus at three-month follow-up.

**Conclusion:**

SARS-CoV-2 infection may plausibly act as a trigger for late-onset MuSK-MG presenting as isolated hypercapnic respiratory failure. In otherwise unexplained weaning failure, neuromuscular-junction disease should be considered even without limb or ocular weakness. Early antibody testing and prompt immunomodulatory therapy [plasma exchange (PLEX) plus glucocorticoid/tacrolimus] may be life-saving and yield rapid recovery.

## Introduction

Myasthenia gravis (MG) with antibodies against MuSK-MG is a subtype of autoimmune neuromuscular junction disorder characterized by the presence of MuSK-Ab (1). Compared to the classic acetylcholine receptor antibody-positive myasthenia gravis (AChR-MG), MuSK-MG predominantly affects muscles innervated by cranial nerves, especially those controlling the pharynx, facial expression, and respiration, while ocular muscles are less frequently involved and limb strength often remains preserved (2, 3). The disease typically has an insidious onset and rapid progression, which can lead to misdiagnosis or delayed diagnosis in the early stages. In severe cases, diaphragmatic paralysis may result in respiratory failure (4).

SARS-CoV-2 infection has been confirmed to trigger various immune-mediated neurological disorders, potentially through such as molecular mimicry, immune reconstitution, or bystander activation (5–7). In recent years, several cases of new-onset MuSK-MG developing within weeks after SARS-CoV-2 infection have been reported. However, cases with delayed onset where respiratory failure is the initial symptom remain rare (8–10).

We report a case of a 29-year-old woman who developed sudden onset of altered consciousness and type II respiratory failure nearly two months after recovering from COVID-19. The etiology was initially unclear despite standard diagnostic evaluations. The diagnosis of MuSK-MG was later confirmed by comprehensive clinical assessment and immunological testing. Although the patient also presented with jaundice, edema, conjunctival congestion, and echocardiographic evidence of severe tricuspid regurgitation, these findings were possibly associated with cardiac dysfunction or other systemic diseases. Notably, respiratory failure was the only symptom definitively attributable to MuSK-MG. This case highlights the importance of considering MuSK-MG in patients presenting with unexplained or isolated respiratory failure during the recovery phase of COVID-19. Early recognition and timely intervention are essential for improving prognosis (11–13).

## Case presentation

A 29-year-old woman, with no known family history or underlying disease, presented with a 6-year history of recurrent chest tightness and shortness of breath. She had visited hospitals several times but remained undiagnosed. Approximately five months ago, she developed bilateral lower limb edema, jaundice, and worsening dyspnea. She was admitted to the Department of Infectious Diseases at the First Affiliated Hospital of Bengbu Medical College. On the third day of admission, she developed respiratory failure and was transferred to the intensive care unit (ICU), where she received endotracheal intubation, anti-infective therapy, and nutritional support.

Upon ICU admission, her temperature was normal, blood pressure was 107/52 mmHg, and heart rate was 112 bpm. Physical examination revealed a systolic murmur in the right cardiac area, bilateral lower limb edema, limited mobility, conjunctival congestion, and dyspnea. Neurological examination showed no obvious abnormalities—no focal motor deficits, dysphagia, curtain sign, or sensory loss. Her difficulty in weaning from mechanical ventilation was initially attributed to right heart failure and liver dysfunction. ICU treatment continued for about one month. These nonspecific symptoms (such as edema, jaundice, conjunctival congestion), combined with echocardiographic findings, suggested that her symptoms were more likely caused by severe tricuspid regurgitation-induced right heart failure and secondary hepatic congestion, rather than directly from others.

Due to the unclear etiology, the patient sought care in Shanghai but was unable to complete further examinations due to financial limitations. Approximately two months ago, she was infected with SARS-CoV-2 and improved with oral antipyretics. She was hospitalized again for further evaluation of previous respiratory symptoms but suddenly lost consciousness while waiting at the outpatient clinic and was urgently transferred to our ICU.

Arterial blood gas analysis indicated type II respiratory failure (PaO_2_: 72.1 mmHg, PaCO_2_: 96.2 mmHg, pH: 7.211, HCO_3_^-^: 38.5 mmol/L), without significant metabolic abnormalities. Chest CT showed mild pneumonia, with no thoracic deformities or thymic lesions. Echocardiography showed severe tricuspid regurgitation and pulmonary hypertension (estimated pressure 68 mmHg). The patient received comprehensive treatment including sildenafil, noninvasive ventilatory support with gradual respiratory function training, nutritional support, methylprednisolone, and respiratory stimulants. Meanwhile, the neostigmine test yielded a negative result. By the third day after admission, the patient’s respiratory function had improved compared with baseline, allowing her to tolerate an off-ward electromyography (EMG) study. The examination showed normal conduction velocities in the bilateral ulnar nerves, the right median nerve, the left tibial nerve, and the bilateral common peroneal nerves, but the compound muscle action potential amplitudes were decreased. No additional abnormalities in conduction velocity, amplitude, or F-wave parameters were detected. Repetitive nerve stimulation (RNS) and single-fiber electromyography (SFEMG) were not performed due to the patient’s critical condition, intolerance to prolonged testing during acute respiratory failure, and the unavailability of SFEMG equipment at our center. Based on clinical data, neuromuscular disease or central nervous system involvement was considered a probable cause of her acute ventilatory failure.

Therefore, the neostigmine test and AChR-Ab assay were repeated on hospital day 5, along with cerebrospinal fluid analysis on day 6; however, all results remained negative. Her symptoms were severe, with a grade IV dyspnea rating based on the modified Medical Research Council (mMRC) scale and a New York Heart Association (NYHA) functional class of immunoglobulin. Due to the lack of a definitive diagnosis from routine investigations, empirical supportive treatment was continued but showed no improvement. Considering the clinical course and presentation, the medical team suspected a rare subtype of myasthenia gravis and sent her serum to a European immunology center for immunofluorescence testing on hospital day 7. The results revealed MuSK-Ab positivity, confirming the diagnosis of MuSK antibody-associated myasthenia gravis on hospital day 9.

**Figure 1:**
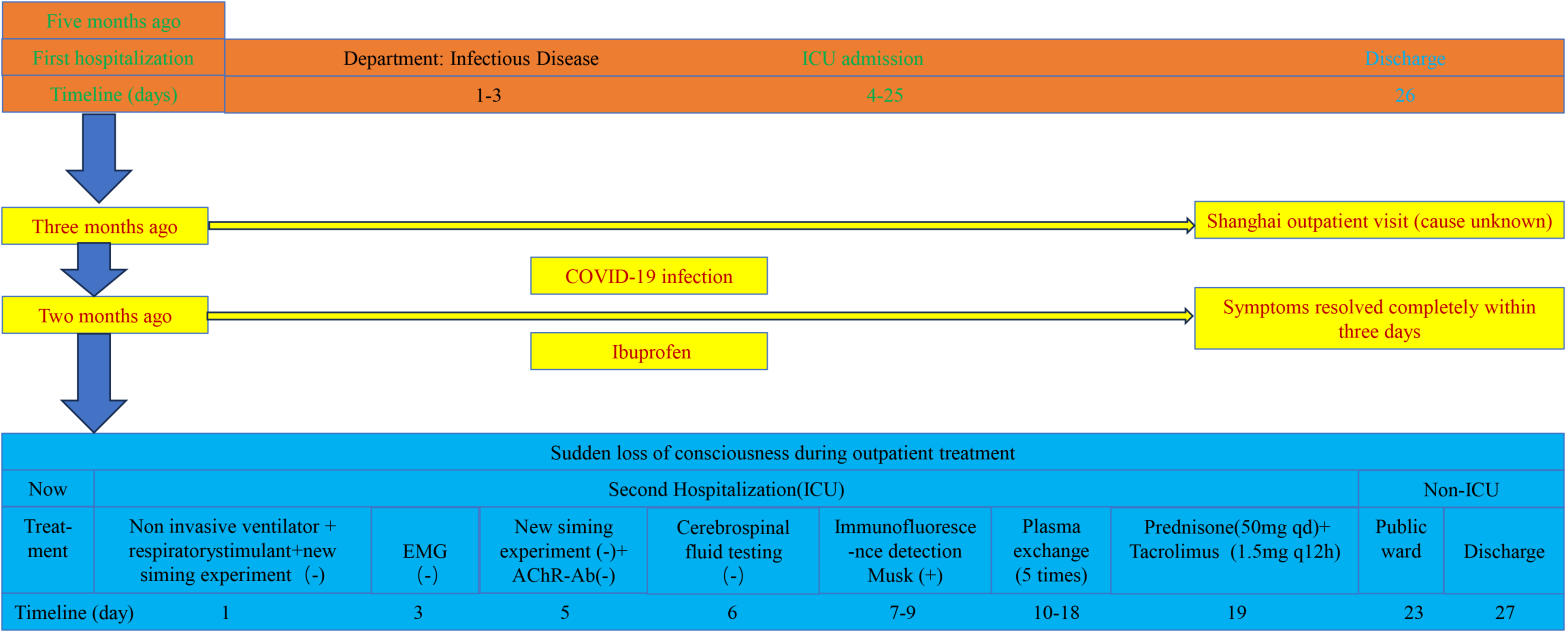
Timeline of clinical course and inpatient management.

Following diagnostic confirmation—and in accordance with current consensus and guideline recommendations for MuSK-MG crisis—we engaged in shared decision-making with the patient and family regarding the efficacy, costs, and risks of available options. We therefore prioritized PLEX, deferred intravenous immunoglobulin (IVIG), and—recognizing its non-acute onset of action—reserved rituximab for later consideration; financial considerations were not the primary factor. The patient underwent five sessions of PLEX from hospital day 10 to day 18, with approximately 3000 mL of plasma exchanged per session. Her dependence on noninvasive ventilation decreased significantly. On day 19, after a joint assessment by the ICU and neurology teams, sequential immunotherapy was initiated, consisting of oral prednisone (50 mg qd), tacrolimus (1.5 mg q12h), and salbutamol (one ampoule via nebulization every 8 hours). Due to financial constraints, IVIG and rituximab were not administered. On treatment day 23, she was successfully weaned off the ventilator, with significant neurological improvement, and was transferred to the general ward. She was discharged on hospital day 27. At discharge, transthoracic echocardiography showed persistent mild tricuspid regurgitation with improved pulmonary artery pressure (decreasing from 68 to 52 mmHg), and lower-limb edema and related symptoms had improved. After respiratory stabilization, RNS was recommended to further characterize neuromuscular transmission; the potential yield and limitations were discussed with the patient, who declined the additional test. At clinic follow-ups, RNS and SFEMG were re-offered to assess neuromuscular transmission, however, given clinical stability and expected limited yield in MuSK-MG, the family again declined; one-year follow-up confirmed stable clinical status with marked symptom remission. A visual timeline of key events is provided in **Figure 1**.

## Discussion

MG with antibodies against MuSK-MG is a rare autoimmune neuromuscular junction disorder, accounting for approximately 5–8% of all MG cases (14, 15). Its pathogenesis involves MuSK-Ab disrupting acetylcholine receptor clustering by interfering with MuSK-mediated signaling, ultimately damaging the postsynaptic membrane and causing muscle weakness (16). Compared with AChR-positive MG, MuSK-MG more often affects cranial nerve-innervated muscles involved in respiration and swallowing, whereas ocular symptoms are relatively mild and limb strength is usually preserved (17, 18). Because its onset is subtle yet progression is rapid, MuSK-MG is often misattributed to cardiopulmonary or systemic conditions, leading to delays in definitive therapy (19, 20).

In the present case, the patient developed MuSK-Ab-positive myasthenia gravis approximately eight weeks after confirmed SARS-CoV-2 infection, with type II respiratory failure as the initial presentation. The absence of other identifiable triggers and the close temporal relationship support a potential triggering role for SARS-CoV-2. COVID-19 has been associated with a range of neuro-immune complications, potentially mediated by mechanisms such as molecular mimicry, bystander activation, or post-infectious immune reconstitution, all of which may result in the production of pathogenic autoantibodies (21, 22). Increasing evidence describes new-onset or exacerbated MG following SARS-CoV-2 infection. MuSK-MG is especially challenging due to its rarity and atypical presentation, such as isolated respiratory failure (23, 24). Cases of newly diagnosed MuSK-Ab-positive myasthenia gravis following SARS-CoV-2 infection have been reported in the literature. Some of these cases represented first-onset presentations, and the majority developed symptoms within several weeks after infection, further supporting a possible triggering association between COVID-19 and MuSK-MG (25, 26). Accordingly, having ruled out other plausible precipitants in our patient, we therefore interpret SARS-CoV-2 as a plausible trigger rather than a demonstrated cause, while explicitly acknowledging the infer.

Early in the present case, signs including bilateral lower limb edema, conjunctival congestion, and mild jaundice, along with echocardiographic findings of severe tricuspid regurgitation and pulmonary hypertension, initially led to a diagnosis of right heart failure and hepatic dysfunction. These were considered the primary causes of weaning failure. However, the sudden development of type II respiratory failure without parenchymal lung involvement or response to anti-infective therapy prompted evaluation for neuromuscular pathology. Subsequent serological testing confirmed the presence of MuSK-Ab, substantiating a neuromuscular basis for the respiratory compromise.

Although AChR-Ab, neostigmine test, and baseline electromyography were negative, neither RNS nor SFEMG was performed due to the patient’s critical condition and inability to tolerate prolonged testing during the ICU phase. Literature reports show that early-stage MuSK-MG may present with negative neostigmine tests and nonspecific EMG results, particularly in cases of selective respiratory muscle involvement (27, 28). Therefore, negative conventional tests should not exclude the diagnosis of MuSK-MG, and serological assays remain pivotal.

Respiratory failure is one of the most life-threatening complications of MuSK-MG, resulting from profound diaphragmatic and intercostal muscle weakness (29, 30). Compared with AChR-MG, MuSK-MG crises are more frequent and severe, with slower recovery and poorer prognosis (31). Although our patient lacked progressive limb weakness, the sudden onset of hypoventilation and hypercapnia highlighted that respiratory crisis can be the initial manifestation of MuSK-MG. Clinicians should maintain a high index of suspicion for MuSK-MG in patients with unexplained or isolated respiratory failure.

Although bulbar predominance is typical in MuSK-MG, selective respiratory involvement with minimal bulbar signs has been described. Several mechanisms may underlie our patient’s presentation (32–35): (i) early, regionally selective vulnerability of the diaphragm/intercostal muscles; (ii) a post-infectious, potentially monophasic course precipitated by SARS-CoV-2; and (iii) inter-individual variability in autoantibody pathogenicity and neuromuscular-junction susceptibility. Compared with published post-COVID-19 MuSK-MG cases, which commonly report bulbar or cranial involvement, our patient’s isolated ventilatory failure at onset appears uncommon (27, 28). The absence of ocular or limb progression over 12 months and sustained response to PLEX/steroids/tacrolimus suggest a limited, possibly monophasic post-infectious phenotype; however, definitive classification is cautioned given the single-case nature.

PLEX remains a cornerstone of acute MuSK-MG management, effectively clearing circulating antibodies and reversing crises (36). In published reports, the majority of MuSK-MG patients presenting with respiratory crises responded favorably to PLEX, especially when initiated early (25, 37, 38). In this case, the patient improved markedly after five sessions of plex (see **Figure 1**). Maintenance therapy with tacrolimus plus low-dose prednisone achieved stable disease control. Tacrolimus inhibits IL-2 transcription in T cells, dampening autoimmune activity and allowing steroid-sparing effects (36). Salbutamol nebulization was added to support neuromuscular transmission and enhance respiratory muscle function (39).

Clinical considerations: In MuSK-MG, acetylcholinesterase inhibitors are often ineffective and may even worsen symptoms; cautious, limited use is advised (40). Thymectomy is not recommended in MuSK-MG without thymoma, and PLEX remains a rapid-acting mainstay for crisis management according to consensus guidance (2). For longer-term control and steroid-sparing, earlier B-cell depletion with rituximab is supported by a 2025 meta-analysis of anti-MuSK MG (41). Rapid clinical improvement can also be achieved via neonatal Fc receptor (FcRn) blockade—for example efgartigimod and rozanolixizumab—with randomized trials demonstrating early benefits in generalized MG cohorts (42, 43). Looking ahead, MuSK-targeted chimeric autoantibody receptor T-cell/CAAR-T (also termed CAART) and chimeric antigen receptor T-cell (CAR-T) represents an investigational direction (44, 45).

In summary, MuSK-MG can manifest with delayed onset following SARS-CoV-2 infection and may present solely as respiratory failure, often misattributed to cardiopulmonary disease. For patients with unexplained hypercapnia and ventilator weaning failure—especially those with recent viral infection—neuromuscular junction disorders should be considered. Early identification, accurate diagnosis, and integrated immunotherapy are vital to improving prognosis in such cases. A visual timeline summarizing the patient’s clinical course and major interventions is presented in **Figure 1**.

## Limitation

(1) As a single-patient report, causality between SARS-CoV-2 and MuSK-MG cannot be established. (2) Electrophysiology was incomplete: RNS/SFEMG were not performed in the ICU due to instability; after stabilization and at follow-up, RNS was re-offered but declined; SFEMG was unavailable. (3) Acute immunotherapy choices followed current consensus—PLEX prioritized, IVIG/rituximab deferred—precluding head-to-head comparisons. (4) Follow-up was limited to 12 months, preventing long-term assessment of relapse and steroid-sparing effects.

## Data Availability

The original contributions presented in the study are included in the article/supplementary material. Further inquiries can be directed to the corresponding author.

## References

[1] Evoli A Alboini PE Damato V Iorio R Provenzano C Bartoccioni E . Myasthenia gravis with antibodies to MuSK: an update. Ann N Y Acad Sci. (2018) 1412:82–9. doi: 10.1111/nyas.13518, PMID: 29266255

[2] Narayanaswami P Sanders DB Wolfe G Benatar M Cea G Evoli A . International consensus guidance for management of myasthenia gravis: 2020 update. Neurology. (2021) 96:114–22. doi: 10.1212/WNL.0000000000011124, PMID: 33144515 PMC7884987

[3] Drachman DB . Myasthenia gravis. Semin Neurol. (2016) 36:419–24. doi: 10.1055/s-0036-1586265, PMID: 27704496

[4] Hoch W McConville J Helms S Newsom-Davis J Melms A Vincent A . Auto-antibodies to the receptor tyrosine kinase MuSK in patients with myasthenia gravis without acetylcholine receptor antibodies. Nat Med. (2001) 7:365–8. doi: 10.1038/85520, PMID: 11231638

[5] Dalakas MC . Guillain-Barré syndrome: The first documented COVID-19-triggered autoimmune neurologic disease: More to come with myositis in the offing. Neurol Neuroimmunol Neuroinflamm. (2020) 7:e781. doi: 10.1212/NXI.0000000000000781, PMID: 32518172 PMC7309518

[6] Li X Wang Y Wang H Wang Y . SARS-CoV-2-associated Guillain-Barré syndrome is a para-infectious disease. QJM. (2021) 114:625–35. doi: 10.1093/qjmed/hcab157, PMID: 34043803 PMC8195029

[7] Toscano G Palmerini F Ravaglia S Ruiz L Invernizzi P Cuzzoni MG . Guillain-barré Syndrome associated with SARS-coV-2. N Engl J Med. (2020) 382:2574–6. doi: 10.1056/NEJMc2009191, PMID: 32302082 PMC7182017

[8] Sriwastava S Tandon M Kataria S Daimee M Sultan S . New onset of ocular myasthenia gravis in a patient with COVID-19: a novel case report and literature review. J Neurol. (2021) 268:2690–6. doi: 10.1007/s00415-020-10263-1, PMID: 33047223 PMC7549728

[9] Restivo DA Centonze D Alesina A Marchese-Ragona R . Myasthenia gravis associated with SARS-coV-2 infection. Ann Intern Med. (2020) 173:1027–8. doi: 10.7326/L20-0845, PMID: 32776781 PMC7429993

[10] Chatterjee T Senthil Kumaran S Roy M . A case report and literature review of new-onset myasthenia gravis after COVID-19 infection. Cureus. (2022) 14:e33048. doi: 10.7759/cureus.33048, PMID: 36721575 PMC9881688

[11] Hehir MK Silvestri NJ . Generalized myasthenia gravis: classification, clinical presentation, natural history, and epidemiology. Neurol Clin. (2018) 36:253–60. doi: 10.1016/j.ncl.2018.01.002, PMID: 29655448

[12] Gilhus NE . Myasthenia gravis. N Engl J Med. (2016) 375:2570–81. doi: 10.1056/NEJMra1602678, PMID: 28029925

[13] Shah SMI Yasmin F Memon RS Jatoi NN Savul IS Kazmi S . COVID-19 and myasthenia gravis: A review of neurological implications of the SARS-COV-2. Brain Behav. (2022) 12:e2789. doi: 10.1002/brb3.2789, PMID: 36306401 PMC9759145

[14] Saied Z Rachdi A Thamlaoui S Nabli F Jeridi C Baffoun N . Myasthenia gravis and COVID-19: A case series and comparison with literature. Acta Neurol Scand. (2021) 144:334–40. doi: 10.1111/ane.13440, PMID: 33914898 PMC8222886

[15] Ali A Almalki D Kotb MA Alenazi RS . Outcomes and characteristics of myasthenia gravis: A 10-year retrospective cross-sectional study at King Fahad Medical City. Neurosci (Riyadh). (2022) 27:237–43. doi: 10.17712/nsj.2022.4.20220038, PMID: 36252965 PMC9749576

[16] Koneczny I Herbst R . Myasthenia gravis: pathogenic effects of autoantibodies on neuromuscular architecture. Cells. (2019) 8:671. doi: 10.3390/cells8070671, PMID: 31269763 PMC6678492

[17] Howard JF Jr Utsugisawa K Benatar M Murai H Barohn RJ Illa I . Safety and efficacy of eculizumab in anti-acetylcholine receptor antibody-positive refractory generalised myasthenia gravis (REGAIN): a phase 3, randomised, double-blind, placebo-controlled, multicentre study. Lancet Neurol. (2017) 16:976–86. doi: 10.1016/S1474-4422(17)30369-1, PMID: 29066163

[18] Di Stefano V Lupica A Rispoli MG Di Muzio A Brighina F Rodolico C . Rituximab in AChR subtype of myasthenia gravis: systematic review. J Neurol Neurosurg Psychiatry. (2020) 91:392–5. doi: 10.1136/jnnp-2019-322606, PMID: 32098874

[19] Bi Z Li Y Lin J Gui M Li Z Bu B . Long-term efficacy and safety of tacrolimus in anti-MuSK antibody-positive myasthenia gravis: a retrospective single-center cohort study. Neurol Sci. (2025) 46:943–9. doi: 10.1007/s10072-024-07819-8, PMID: 39503950

[20] Baheerathan A Dorsey R Viegas S . Oral salbutamol for symptomatic treatment in MuSK antibody-positive myasthenia gravis: a single-centre experience. Acta Neurol Belg. (2024) 124:1737–8. doi: 10.1007/s13760-024-02541-w, PMID: 38561498

[21] Zhao S Zhang K Ren K Lu J Ma C Zhao C . Clinical features, treatment and prognosis of MuSK antibody-associated myasthenia gravis in Northwest China: a single-centre retrospective cohort study. BMC Neurol. (2021) 21:428. doi: 10.1186/s12883-021-02439-7, PMID: 34732168 PMC8567678

[22] Carr AS Cardwell CR McCarron PO McConville J . A systematic review of population based epidemiological studies in Myasthenia Gravis. BMC Neurol. (2010) 10:46. doi: 10.1186/1471-2377-10-46, PMID: 20565885 PMC2905354

[23] Zain A Akram MS Ashfaq F Ans A Ans HH . Comparative analysis of intravenous immunoglobulins (IVIg) vs plasmapheresis (PLEX) in the management of myasthenic crisis. Cureus. (2024) 16:e68895. doi: 10.7759/cureus.68895, PMID: 39376877 PMC11458158

[24] Crisafulli S Boccanegra B Carollo M Bottani E Mantuano P Trifirò G . Myasthenia gravis treatment: from old drugs to innovative therapies with a glimpse into the future. CNS Drugs. (2024) 38:15–32. doi: 10.1007/s40263-023-01059-8, PMID: 38212553

[25] Jha S Pendyala SK . MuSK antibody-positive myasthenia gravis with SARS CoV-2 infection: a case report and literature review. J Clin Neuromuscul Dis. (2024) 25:203–5. doi: 10.1097/CND.0000000000000491, PMID: 38771234

[26] Sanders DB Arimura K Cui L Ertaş M Farrugia ME Gilchrist J . Guidelines for single fiber EMG. Clin Neurophysiol. (2019) 130:1417–39. doi: 10.1016/j.clinph.2019.04.005, PMID: 31080019

[27] Guidon AC Amato AA . COVID-19 and neuromuscular disorders. Neurology. (2020) 94:959–69. doi: 10.1212/WNL.0000000000009566, PMID: 32284362

[28] Camdessanche JP Morel J Pozzetto B Paul S Tholance Y Botelho-Nevers E . COVID-19 may induce Guillain-Barré syndrome. Rev Neurol (Paris). (2020) 176:516–8. doi: 10.1016/j.neurol.2020.04.003, PMID: 32334841 PMC7158797

[29] Gilhus NE Verschuuren JJ . Myasthenia gravis: subgroup classification and therapeutic strategies. Lancet Neurol. (2015) 14:1023–36. doi: 10.1016/S1474-4422(15)00145-3, PMID: 26376969

[30] Sieb JP . Myasthenia gravis: an update for the clinician. Clin Exp Immunol. (2014) 175:408–18. doi: 10.1111/cei.12217, PMID: 24117026 PMC3927901

[31] Gerischer L Doksani P Hoffmann S Meisel A . New and emerging biological therapies for myasthenia gravis: A focussed review for clinical decision-making. BioDrugs. (2025) 39:185–213. doi: 10.1007/s40259-024-00701-1, PMID: 39869260 PMC11906560

[32] Rodolico C Bonanno C Toscano A Vita G . MuSK-associated myasthenia gravis: clinical features and management. Front Neurol. (2020) 11:660. doi: 10.3389/fneur.2020.00660, PMID: 32793097 PMC7390870

[33] Doumiati H Ezzeddine A . Isolated respiratory failure as the presenting symptom of muscle-specific kinase myasthenia gravis: a case report and literature review. Case Rep Neurol. (2024) 16:233–41. doi: 10.1159/000540916, PMID: 39474296 PMC11521485

[34] Wang F Cheng J Niu X Li L . Respiratory failure as first presentation of myasthenia gravis: a case report. J Int Med Res. (2024) 52:3000605241234585. doi: 10.1177/03000605241234585, PMID: 38443765 PMC10916481

[35] Assini A Gandoglia I Damato V Rikani K Evoli A Del Sette M . Myasthenia gravis associated with anti-MuSK antibodies developed after SARS-CoV-2 infection. Eur J Neurol. (2021) 28:3537–9. doi: 10.1111/ene.14721, PMID: 33421278 PMC8014563

[36] Berrih-Aknin S . Myasthenia Gravis: paradox versus paradigm in autoimmunity. J Autoimmun. (2014) 52:1–28. doi: 10.1016/j.jaut.2014.05.001, PMID: 24934596

[37] Mincă A Mincă DI Calinoiu AL Gheorghiţă V Popescu CC Rusu A . Myasthenia gravis triggered by a COVID-19 infection: A case report and literature review. Cureus. (2024) 16:e59538. doi: 10.7759/cureus.59538, PMID: 38827012 PMC11144031

[38] Usmani A Kwan L Wahib-Khalil D Trivedi J Nations S Sarode R . Excellent response to therapeutic plasma exchange in myasthenia gravis patients irrespective of antibody status. J Clin Apher. (2019) 34:416–22. doi: 10.1002/jca.21694, PMID: 30779438

[39] Hurst RL Gooch CL . Muscle-specific receptor tyrosine kinase (MuSK) myasthenia gravis. Curr Neurol Neurosci Rep. (2016) 16:61. doi: 10.1007/s11910-016-0668-z, PMID: 27170368

[40] Ricciardi R Latini E Guida M Koneczny I Lucchi M Maestri M . Acetylcholinesterase inhibitors are ineffective in MuSK-antibody positive myasthenia gravis: Results of a study on 202 patients. J Neurol Sci. (2024) 461:123047. doi: 10.1016/j.jns.2024.123047, PMID: 38759248

[41] Chayanopparat S Banyatcharoen P Jitprapaikulsan J Uawithya E Apiraksattayakul N Viarasilpa V . Efficacy and safety of rituximab in anti-MuSK myasthenia Gravis: a systematic review and meta-analysis. Sci Rep. (2025) 15:7219. doi: 10.1038/s41598-025-90937-w, PMID: 40021769 PMC11871026

[42] Howard JF Jr Bril V Vu T Karam C Peric S Margania T . Safety, efficacy, and tolerability of efgartigimod in patients with generalised myasthenia gravis (ADAPT): a multicentre, randomised, placebo-controlled, phase 3 trial. Lancet Neurol. (2021) 20:526–36. doi: 10.1016/S1474-4422(21)00159-9, PMID: 34146511

[43] Bril V Drużdż A Grosskreutz J Habib AA Mantegazza R Sacconi S . Safety and efficacy of rozanolixizumab in patients with generalised myasthenia gravis (MycarinG): a randomised, double-blind, placebo-controlled, adaptive phase 3 study. Lancet Neurol. (2023) 22:383–94. doi: 10.1016/S1474-4422(23)00077-7, PMID: 37059507

[44] Binks SNM Morse IM Ashraghi M Vincent A Waters P Leite MI . Myasthenia gravis in 2025: five new things and four hopes for the future. J Neurol. (2025) 272:226. doi: 10.1007/s00415-025-12922-7, PMID: 39987373 PMC11846739

[45] Oh S Mao X Manfredo-Vieira S Lee J Patel D Choi EJ . Precision targeting of autoantigen-specific B cells in muscle-specific tyrosine kinase myasthenia gravis with chimeric autoantibody receptor T cells. Nat Biotechnol. (2023) 41:1229–38. doi: 10.1038/s41587-022-01637-z. Erratum in: Nat Biotechnol. 2024 Dec;42(12):1923. doi: 10.1038/s41587-024-02502-x., PMID: 36658341 PMC10354218

